# Unexpected reaction of “wild-type” gastrointestinal stromal tumor to imatinib: case report and literature review

**DOI:** 10.3389/fonc.2023.1334784

**Published:** 2024-01-31

**Authors:** Yang He, Mingxu Da, Chuanlei Fan, Pengxian Tao

**Affiliations:** ^1^ The First Clinical Medical College, Gansu University of Chinese Medicine, Lanzhou, China; ^2^ Department of Surgical Oncology, Gansu Provincial Hospital, Lanzhou, China; ^3^ The First Clinical Medical College, Lanzhou University, Lanzhou, China; ^4^ Department of General Surgery Cadre Ward, Gansu Provincial Hospital, Lanzhou, China

**Keywords:** gastrointestinal stromal tumors, neoadjuvant therapy, imatinib, laparoscopic surgery, case report

## Abstract

**Background:**

Most of gastrointestinal stromal tumors (GISTs) are driven by mutations in the KIT/PDGFRA genes and can benefit from TKIs treatment. However, a small subset of GIST (10%-15%) are called “wild-type” GISTs due to the lack of these mutations. Theoretically, they would not benefit from TKIs treatment and may even develop resistance. Therefore, this unexpected response may challenge inherent perceptions. Herein, we present a case of giant wild-type GIST exhibiting an unexpected response to imatinib(IM), followed by laparoscopic surgical resection. Subsequently, potential underlying mechanisms are discussed.

**Case description:**

This case describes a 57-year-old man who presented with abdominal pain for two weeks. CT revealed a massive lesion near the splenic hilum along the greater curvature of the stomach, concurrently involving the splenic hilar vessels and surrounding lymph nodes. Ultrasound-guided fine needle aspiration biopsy confirmed it is a mesenchymal spindle cell tumor,GIST. Due to the enormous volume and local invasion, neoadjuvant chemotherapy was initially considered. After 6 months of IM 400 mg/d, CT imaging revealed marked changes in tumor heterogeneity and a significant reduction in volume. Subsequently, laparoscopic surgical resection was performed. Postoperative pathological examination, immunohistochemistry, and genetic testing collectively confirmed it is a wild-type GIST.The patient recovered well and was discharged on the 6th day after surgery, with continued oral IM(400 mg/d) after discharge. No recurrence was observed during follow-up until the publication of this report.

**Conclusion:**

This unexpected response suggests that wild-type GISTs may benefit from TKIs treatment, and the potential mechanisms warrant further investigation. Additionally, true wild-type GIST may not be discerned due to current limitations of Next-Generation Sequencing(NGS). Therefore, for advanced/high-risk GIST, additional genetic analysis can be performed after negative NGS results.

## 1 Introduction

90% of gastrointestinal stromal tumor (GISTs) are driven by mutually exclusive mutations in c-kit (KIT) and platelet-derived growth factor receptor alpha (PDGFRA) (1, 2). Both belong to the tyrosine kinase family III receptors and share similar structures; any mutation in either gene leads to a conformational change in the ATP-binding domain (3). Mutations in KIT and PDGFRA activate downstream signaling pathways in a similar manner, including MAPK, AKT, STAT1, and STAT3 pathways, leading to uncontrolled cell proliferation (4, 5).

Generally, tyrosine kinase inhibitors (TKIs), such as imatinib(IM), competitively occupy the ATP binding site, preventing signaling pathways activation and extending the overall survival of patients. However, a small subset of GISTs (10%-15%) are called “wild-type” GISTs due to the lack of these mutations (6). It also explains why wild-type GISTs are typically insensitive to TKIs treatment. Based on cases exhibiting unexpected reactions to IM, we explore the potential mechanisms through which wild-type GISTs may benefit from IM treatment.

## 2 Case description

**Figure 1:**
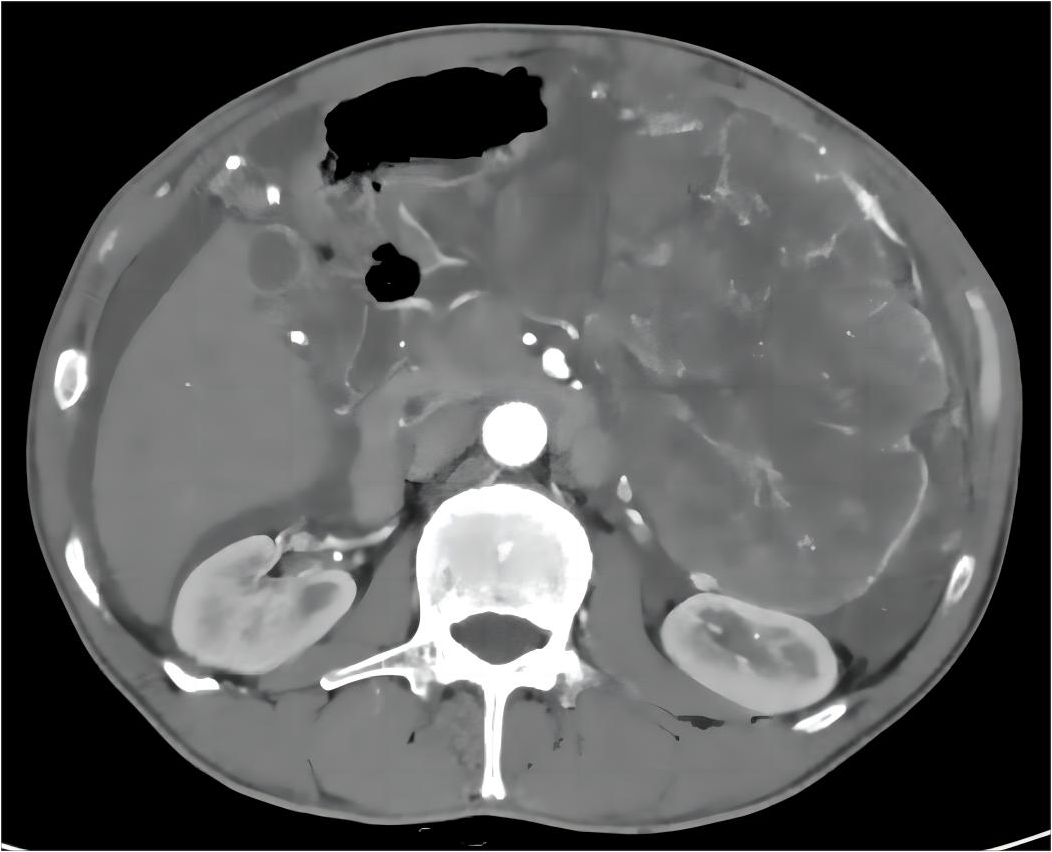
Pre-treatment abdominal CT results with imatinib: A massive tumor on the left side of the abdomen.

This study describes a 57-year-old man who presented with abdominal pain for two weeks. This was the sequence of events in the hospital (**Supplementary Figure 1**). Physical examination revealed a massive mass measuring approximately 10cm×10cm in the left upper abdomen. The mass exhibited a hardened texture and limited mobility. Laboratory tests revealed a gradual increase in NLR, PLR, and CRP over time, accompanied by a gradual decrease in Alb. This was the trend of these indicators over time (**Supplementary Figure 2**). Surprisingly, the patient did not exhibit any other discomfort. CT imaging (**Figure 1**) revealed a massive tumor near the splenic hilum in the greater curvature of the stomach (162.9×113.2×217.7 mm³), exerting pressure on nearby vessels and organs. Meanwhile, the tumor invaded both the splenic hilar vessels and surrounding lymph nodes. Ultrasound-guided fine needle aspiration biopsy confirmed it is a mesenchymal spindle cell tumor, GIST (**Supplementary Figure 3**). Due to the extensive volume and local invasion, neoadjuvant chemotherapy was initially considered.

**Figure 2:**
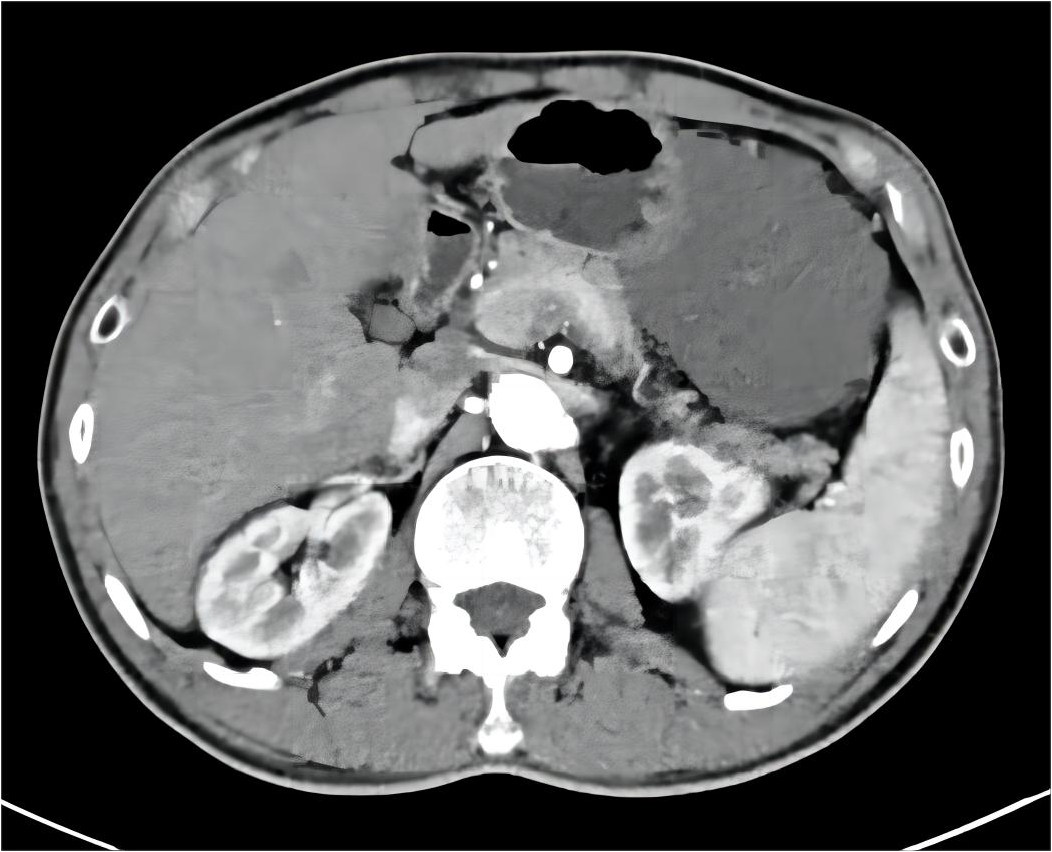
Post-treatment abdominal CT results with imatinib: the primary tumor is significantly reduced.

**Figure 3:**
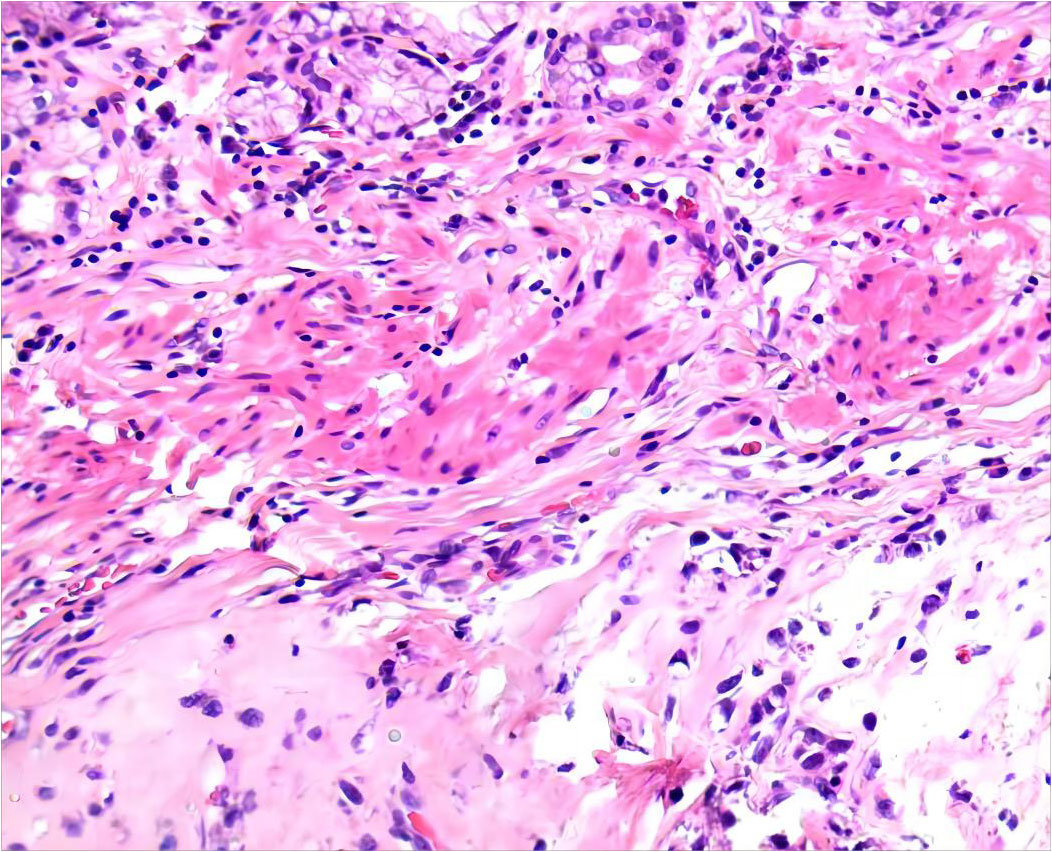
Postoperative pathological (10*10 HE): gastrointestinal stromal tumor (GIST).

After 6 months of IM 400 mg/day, tumor volume was reduced by 81.68% (93.8×70.2×111.7 mm³) compared to previous CT results, calculated using the clinical volume formula v = 1/2abc (7). Meanwhile, tumor heterogeneity was markedly altered (**Figure 2**). Laparoscopic resection was subsequently performed. Postoperative pathological examination confirmed a spindle cell tumor of mesenchymal origin, GIST. The mitosis was 6/50 high power field (**Figure 3**). According to the NIH classification (8), Modified NIH classification (9), AFIP classification (10), Glasgow Prognostic Score (11), it is classified as a high-risk GIST. Immunohistochemical revealed CKP(-); Vimentin(+); Desmin(-); SMA(+); CD34(+); CD117(+); DOG-1(+); S-100(-); SDHB(+); Ki-67(index:20%) (**Supplementary Figure 4**). No C-kit 9/11/13/17 and PDGPRA 12/18 mutations were detected by the genetic test. The patient recovered well and was discharged on the 6th day after surgery, with continued oral IM (400 mg/d) after discharge. No recurrence was observed during follow-up until the publication of this report. The patient was compliant and well tolerated throughout the course of treatment with no adverse events. The upcoming article will refer to the CARE report list for further discussion.

## 3 Discussion

We first report a case of a large and locally advanced wild-type GIST patient who significantly benefited from IM treatment, followed by subsequent laparoscopic resection. This unexpected result suggests that neoadjuvant chemotherapy combined with laparoscopic surgery may be a promising strategy. According to the recommendations, additional genetic analysis should be conducted for confirmation, as accurate diagnosis is crucial for the treatment and prognosis of patients (12). Unfortunately, due to certain reasons, it was not possible to proceed. Since the discovery that GISTs is driven by c-kit mutations, there has been a revolutionary change in our understanding of its genetic mechanisms. Although theoretically wild-type GISTs should not benefit from IM therapy, in reality, approximately 30% of patients achieve positive outcomes (13). Building on this scenario, we conducted a literature review in an effort to identify the theoretical basis for the unexpected response.

Unknown mutations may potentially exist, contributing to the benefit of wild-type GISTs from IM therapy. The primary mutations in the KIT/PDGFRA genes are mutually exclusive, yet they can coexist with secondary mutations in the KIT/PDGFRA genes (14). This implies the concurrent presence of both primary and secondary mutations in the KIT/PDGFRA genes (12). Jing et al. confirmed the coexistence of wild-type and mutant tumor cells in 4 GIST patients with KIT mutations. This observation may trigger polyclonal evolution and result in unique treatment sensitivities (13). Mechanistically, GIST cells with KIT 11 or 9 mutations developed *de novo* secondary mutations in KIT 13/14/17/18 following IM treatment. Additionally, Bombac et al. identified a rare case of double-mutant GISTs, with two distinct primary mutations within PDGFRA 14 (15). These potential mutation sites make it possible for wild-type GIST patients to benefit from IM therapy. Therefore, wild-type GIST patients may benefit from unknown mutations. For instance, in addition to common 9/11/13/17 mutations, GIST cells with 8/10 mutations in the KIT gene exhibit sensitivity to IM therapy (16). KIT protein is overexpressed in 90% of GIST cases, yet it does not directly correlate with KIT gene mutations (14). Our immunohistochemistry demonstrated KIT protein expression, but genetic testing did not detect c-kit 9/11/13/17 mutations. Apart from objective factors such as technical issues and sample quality, this result implies the potential existence of undetected exon mutations in Kit.

Additionally, wild-type GISTs carry multiple gene mutations. Hechtman et al. conducted sequencing on wild-type GIST patients and identified SDHB deletion in all patients, accompanied by mutations in other genes such as ARID1A and TP53 (17). In patients with wild-type GIST, Agaimy et al. identified BRAF mutations(7%) (12), while another study by Gao et al. revealed an equal incidence of BRAF and KRAS mutations(1.7%) (13). Additionally, EGFR mutations have also been reported in wild-type GISTs (14). For quadruple-negative GISTs lacking KIT/PDGFRA/SDH/RAS mutations, various studies have also detected a common NF1 mutation (18, 19). Despite variations in mutation frequencies, experimental evidence confirms that wild-type GISTs carry multiple mutations with unknown functional effects. Further research is needed to investigate the response of these mutated genes in IM therapy. Due to limitations in Next-Generation Sequencing(NGS) technology, other potentially pathogenic sequences may not be found. Mathilde et al. reported a positive response to IM treatment in a wild-type GIST patient. Genetic analysis showed pathogenic 45-bp repeats found in tumor cells, which unfortunately could not be detected by traditional sequencing (20).

Studies indicate a high infiltration of immune cells in GIST, prompting us to shift our focus towards the immune system and the tumor microenvironment(TME) to seek answers. Wild-type GIST patients may benefit from off-target effects of IM. TME plays a crucial role in the process of tumor evolution (21). In GISTs, the TME is primarily composed of tumor-associated macrophages (TAMs) and T cells, with TAMs outnumbering T cells approximately 2-fold (22). CD8+ T cells, as pivotal cells in anti-tumor immunity, are highly expressed too. Additionally, there are natural killer cells (NK cells), dendritic cells (DCs), and regulatory T(Treg) cells (23). These components collectively contribute to the complex interplay within TME, influencing the dynamics of tumor malignancy. Based on the density of immune cell infiltration, we discussed the potential mechanisms of imatinib-mediated off-target effects.

TAM is one of the most abundant inflammatory cells in the TME and can be divided into M1 and M2 subtypes, with opposing functions. In untreated primary GISTs, there is still controversy regarding the polarization tendency of macrophages, and IM may exacerbate this dual difference (22). Different studies elucidate various polarization pathways of TAMs. Research has reported that the number of macrophages in metastatic lesions is twice that of the primary lesions. Therefore, TAMs are often described as M2-polarized, thereby promoting immune suppression (22, 24). However, Francesca et al. reached the opposite conclusion. They found that IM promotes the transition from M2 macrophages to M1 macrophages through Toll-like Receptor, not only restoring their immune function but also promoting the release of immune cytokines and activation of NK cells (25). Cavnar et al. discovered that the status of TAMs is dynamic. TAMs exhibit a phenotype and function similar to M1 macrophages at baseline, IM promotes TAMs polarization towards M2 macrophages, and in imatinib-resistant GIST, TAMs revert to their original phenotype and gene expression profile. It involves the interaction between TAMs and apoptotic tumor cells, subsequently inducing the expression of CCAT-enhancer binding protein b transcription factor (26). Van et al.’s study supports this effect. IM increases IL-10 secretion by M1 macrophages and reduces the generation of Treg cells to promote anti-tumor effects (14). Therefore, the status of TAMs may be a key factor in the benefit of wild-type GISTs from IM.

T lymphocytes primarily consist of CD3^+^ T, CD8^+^ T, and Treg cells (14). In GISTs, as a critical component of tumor immunity, the infiltration of CD8^+^ T cells affects patient Recurrence-Free Survival (27). Multiple studies have found that nearly all GISTs exhibit high-density infiltration of CD8^+^ T cells and are associated with various factors (28). For instance, in KIT/PDGFRA-deficient GISTs, the quantity of CD8^+^ T cells is significantly higher than in mutated types (27). Compared to untreated and imatinib-resistant GISTs, imatinib-sensitive GISTs exhibit an increased infiltration density of CD3^+^ T and CD8^+^ T cells, while the infiltration density of Treg cells decreases (14). Apart from that, researchers are investigating the influence of IM on T cells in GIST mice. Hirata et al. confirmed that IM enhances the expression of IFN-γ in CD8^+^ T cells, increases the infiltration of CD8^+^ T cells in mice, thereby inducing anti-tumor immunity (29). Balachandran et al. reported that IM suppresses indoleamine 2,3-dioxygenase(IDO) expression by inhibiting KIT signaling, thereby altering the immune function. On one side, IM amplifies the immune effects of pre-existing CD8^+^ T cells. Specifically, IM induces the proliferation and cytolytic capability of CD8^+^ T cells, thereby enhancing immune effects. On the other side, IM induces apoptosis and diminishes the infiltration density of Treg cells, while concurrently upregulating the CD8^+^T/Treg ratio (30). It has been demonstrated that the decline in CD8^+^ T/Treg ratio is a major factor in immune escape (14). However, Tieniber et al.’s study indicated that short-term treatment with IM increases the infiltration density of DCs and CD8^+^ T cells in tumors, promoting DCs maturation. In contrast, long-term treatment yields the opposite effect (30).

NK cells, as an integral component of the human innate immune system, represent a crucial subset of cytotoxic effector cells (14). The degree of NK cell infiltration in GISTs is positively correlated with patient prognosis (24). NK cells target cells with low MHC-I expression, serving as a vital complement to cellular immunity. Similar to CD8^+^ T cells, NK cell infiltration is also associated with genetic mutations, with the number of NK cells in KIT-deficient GISTs being three times higher than that in mutated types (31). KIT protein is expressed on the surface of DCs and participates in the cross-activation of NK cells. Tan et al. revealed that IM directly binds to the cell surface KIT protein receptor, inducing NK cell activation and enhancing Th1 immune response. This promotes interaction between DCs and NK cells, increasing NK cell secretion of IFN-γ, thereby achieving a more potent anti-tumor effect (32). Bellora et al. confirmed that IM, by downregulating CXCR3 and upregulating CXCR4 chemokine receptor, promotes the repositioning of NK cells to the primary site, enhancing immune surveillance against tumor cells (33).

Therefore, for advanced/high-risk GISTs, additional genetic analysis can be performed after the NGS result is negative, because accurate diagnosis is very important for the treatment and prognosis of patients. In addition, we conducted a literature review on wild-type GISTs and explored potential mechanisms underlying unexpected responses. Theoretically, wild-type GISTs should not derive benefit from IM treatment. However, beyond focusing solely on the genetic mutation perspective, IM may achieve these unexpected responses by modulating the TME and immune cells. Ultimately,TME and immune cells may represent crucial directions for further research to deepen our understanding of the pathogenesis of wild-type GISTs and identify more effective therapeutic strategies.

## Patient perspective

Name: Wei (fictitious name)

Age: 57 years

Gender: Male

Diagnosis: Wild type gastrointestinal stromal tumor

I‘m Wei, a 57-year-old farmer. About 2 weeks ago, I had abdominal pain and loss of appetite. It didn‘t get my enough attention at first, thinking it’s just indigestion. However, as the abdominal pain continued to worsen, I began to feel a flash of panic. So, on 25 Apr 2022, I came to the doctor for help and performed a series of subsequent tests. CT contrast on 27 Apr 2022 confirmed a large mass in my abdomen; ultrasound guided fine needle aspiration biopsy on 05 May 2022 confirmed it to be a gastrointestinal stromal tumor. It was not until then that I realized that the condition was very serious, full of fear, and began to worry about my future.

At the doctor‘s advice, I received oral imatinib 400 mg/d. Fortunately, I was relieved that there were no drug side effects and no physical discomfort that bothered me.

After taking imatinib for 6 months, I performed CT contrast again on 30 Dec 2022. To my delight, the mass had significantly decreased in size, which meant that it could be surgically removed. I subsequently underwent laparoscopic surgical resection. On January 3, 2023, Postoperative pathological examination, immunohistochemistry and genetic testing jointly confirmed that it was wild-type GIST. This result makes my doctor happy and puzzled because wild-type GISTs usually do not benefit from imatinib treatment.

Now I do CT every 3 months, so far there is no recurrence, although the probability of recurrence of gastrointestinal stromal tumors is not low. Slowly, I learned to accept and face reality. I hope my story can bring hope to other patients, strong face of disease, cherish every moment of life.

Above are my personal feelings and experiences in the treatment of giant gastrointestinal stromal tumors in the abdominal cavity, thank you.

## Data availability statement

The original contributions presented in the study are included in the article/**Supplementary Material**, further inquiries can be directed to the corresponding author.

## Ethics statement

The studies involving humans were approved by he Medical Ethics Committee of Gansu Provincial People’s Hospital (No: 2023-388). The studies were conducted in accordance with the local legislation and institutional requirements. The participants provided their written informed consent to participate in this study. Written informed consent was obtained from the individual(s) for the publication of any potentially identifiable images or data included in this article.

## Author contributions

YH: Conceptualization, Data curation, Investigation, Writing-original draft, Writing-review & editing. CF: Data curation, Writing-review & editing. PT: Supervision, Validation, Writing-review & editing. MD: Supervision, Validation, Writing-review & editing.

## Supplementary material

Supplementary Figure 1 This is the sequence of events in the hospital.

Supplementary Figure 2 Changes in NLR, PLR, CRP and Alb over time.

Supplementary Figure 3 Ultrasound-guided fine needle aspiration biopsy result.

Supplementary Figure 4 IHC showing: A:40*10 CD34 B:40*10 Dog-1 C:40*10 CD117.
