# Supplementary material for: Unexpected reaction of “wild-type” gastrointestinal stromal tumor to imatinib: case report and literature review
Source: Front Oncol. 2024 Jan 31;13:1334784. doi: 10.3389/fonc.2023.1334784 (PMC10864548; doi:10.3389/fonc.2023.1334784)
Supplement: Supplementary Figure 1 — This is the sequence of events in the hospital. [file DataSheet_1.zip › Supplementary_Material 123.docx]

Supplementary Figures


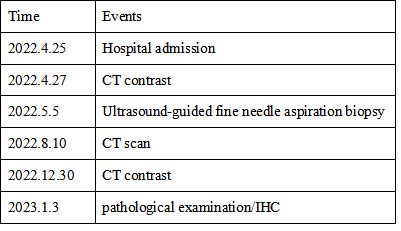


**Supplementary Figure 1.** This is the sequence of events in the hospital.


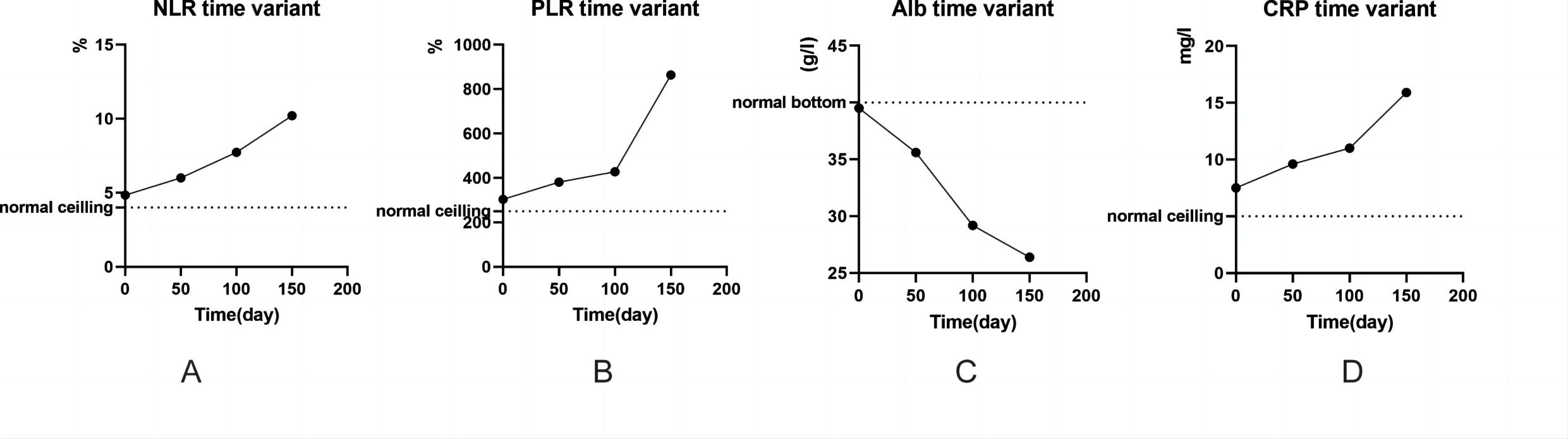


**Supplementary Figure 2.** Changes in NLR, PLR, CRP and Alb over time.


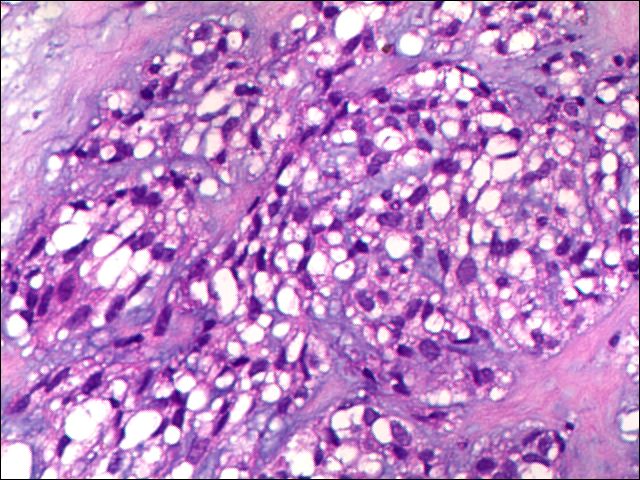


**Supplementary Figure 3.** Ultrasound-guided fine needle aspiration biopsy result.


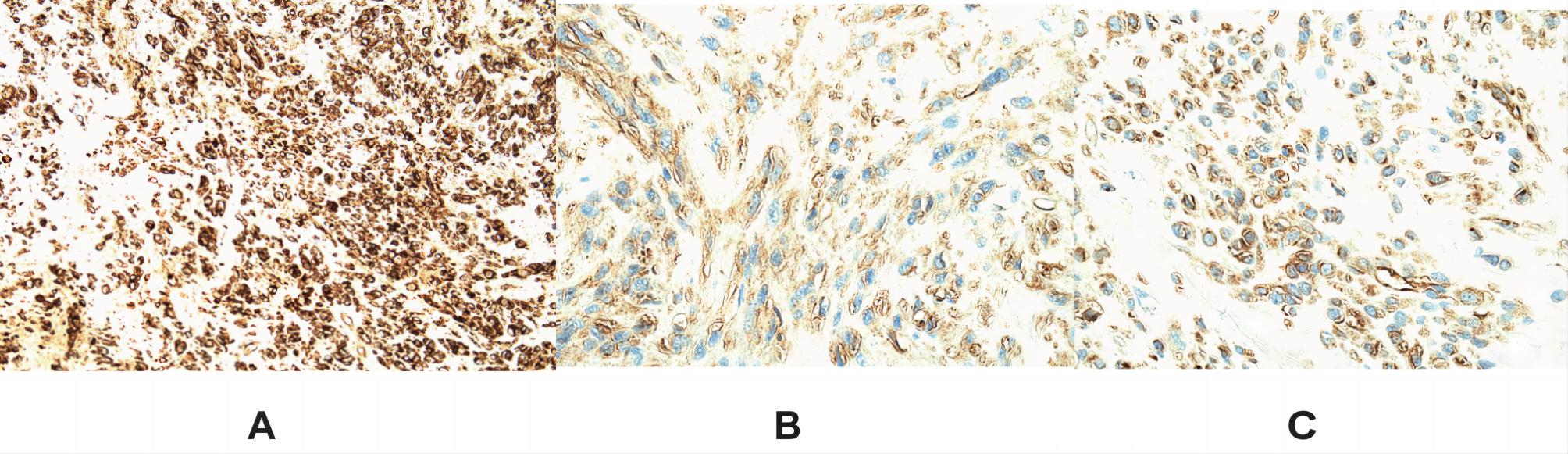


**Supplementary Figure 4.** IHC showing: A：40*10 CD34 B:40*10 Dog-1 C:40*10 CD117
