# Supplementary figures and images for: Unexpected reaction of “wild-type” gastrointestinal stromal tumor to imatinib: case report and literature review
Source: Front Oncol. 2024 Jan 31;13:1334784. doi: 10.3389/fonc.2023.1334784 (PMC10864548; doi:10.3389/fonc.2023.1334784)

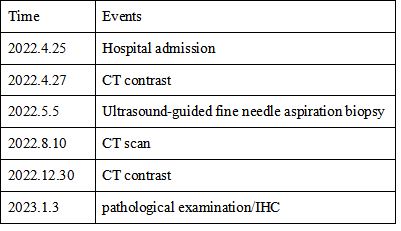

Supplement: Supplementary Figure 1 — This is the sequence of events in the hospital. [file DataSheet_1.zip › Supplementary 1.JPG]

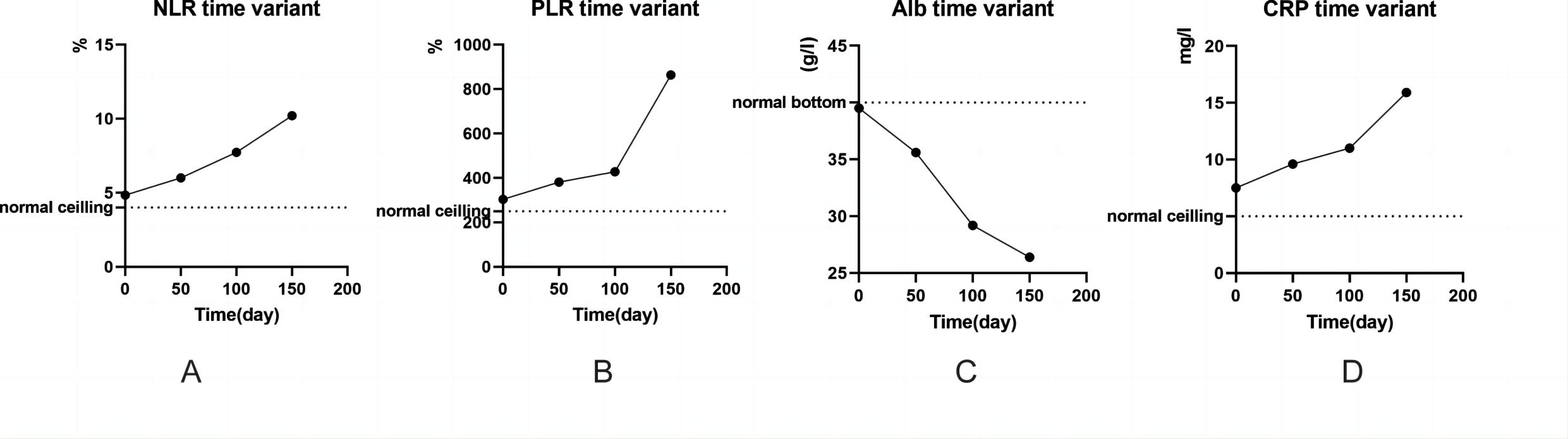

Supplement: Supplementary Figure 1 — This is the sequence of events in the hospital. [file DataSheet_1.zip › Supplementary 2.jpg]

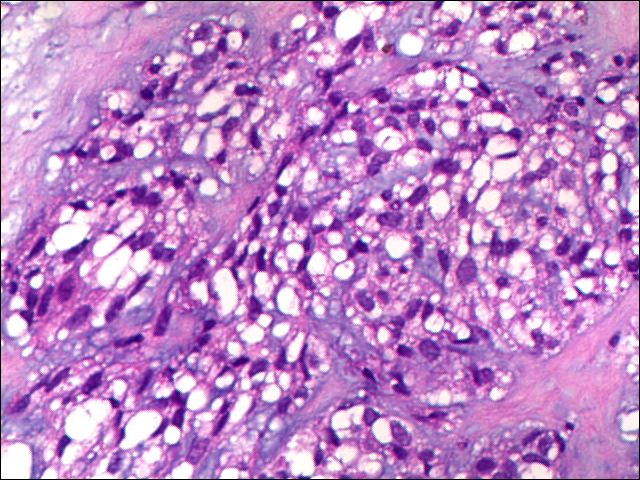

Supplement: Supplementary Figure 1 — This is the sequence of events in the hospital. [file DataSheet_1.zip › Supplementary 3.jpg]

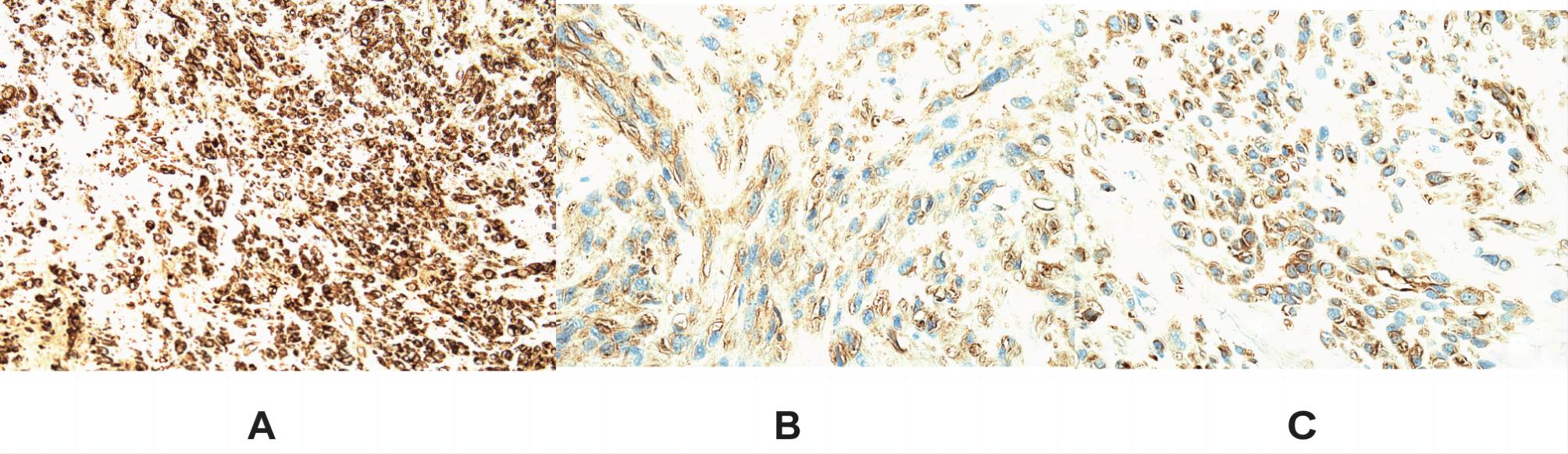

Supplement: Supplementary Figure 1 — This is the sequence of events in the hospital. [file DataSheet_1.zip › Supplementary 4.jpg]
